# Trypanosomatid Richness Among Rats, Opossums, and Dogs in the Caatinga Biome, Northeast Brazil, a Former Endemic Area of Chagas Disease

**DOI:** 10.3389/fcimb.2022.851903

**Published:** 2022-06-20

**Authors:** Maria Augusta Dario, Carolina Furtado, Cristiane Varella Lisboa, Felipe de Oliveira, Filipe Martins Santos, Paulo Sérgio D’Andrea, André Luiz Rodrigues Roque, Samanta Cristina das Chagas Xavier, Ana Maria Jansen

**Affiliations:** ^1^ Trypanosomatid Biology Laboratory, Oswaldo Cruz Institute, Oswaldo Cruz Foundation, Rio de Janeiro, Brazil; ^2^ Genetic Laboratory, National Cancer Institute, Rio de Janeiro, Brazil; ^3^ Environmental Sciences and Agricultural Sustainability Postgraduation, Dom Bosco Catholic University, Campo Grande, Brazil; ^4^ Wild Mammal Reservoirs Biology and Parasitology Laboratory, Oswaldo Cruz Institute, Oswaldo Cruz Foundation, Rio de Janeiro, Brazil

**Keywords:** Trypanosomatidae richness, *Trypanosoma cruzi* infection, *T. cruzi* DTU TcI haplotype, synantropic mammals, *Canis familiaris*, Caatinga biome

## Abstract

Parasites are important components of the immense n-dimensional trophic network that connects all living beings because they, among others, forge biodiversity and deeply influence ecological evolution and host behavior. In this sense, the influence of Trypanosomatidae remains unknown. The aim of this study was to determine trypanosomatid infection and richness in rats, opossums, and dogs in the semiarid Caatinga biome. We submitted DNA samples from trypanosomatids obtained through axenic cultures of the blood of these mammals to mini exon multiplex-PCR, Sanger, and next-generation sequencing targeting the 18S rDNA gene. Phylogenetic analyses were performed to identify genetic diversity in the Trypanosomatidae family. Shannon, Simpson, equability, and beta-diversity indices were calculated per location and per mammalian host. Dogs were surveyed for trypanosomatid infection through hemocultures and serological assays. The examined mammal species of this area of the Caatinga biome exhibited an enormous trypanosomatid species/genotypes richness. Ten denoised Operational Taxonomic Units (ZOTUs), including three species (*Trypanosoma cruzi*, *Trypanosoma rangeli* and *Crithidia mellificae*) and one *Trypanosoma* sp. five genotypes/lineages (*T. cruzi* DTU TcI, TcII, and TcIV; *T. rangeli* A and B) and four DTU TcI haplotypes (ZOTU1, ZOTU2, ZOTU5, and ZOTU10 merged), as well as 13 Amplicon Sequence Variants (ASVs), including five species (*T. cruzi*, *T. rangeli*, *C. mellificae*, *Trypanosoma dionisii*, and *Trypanosoma lainsoni*), five genotypes/lineages (same as the ZOTUs) and six DTU TcI haplotypes (ASV, ASV1, ASV2, ASV3, ASV5 and ASV13), were identified in single and mixed infections. We observed that trypanosomatids present a broad host spectrum given that species related to a single host are found in other mammals from different taxa. Concomitant infections between trypanosomatids and new host-parasite relationships have been reported, and this immense diversity in mammals raised questions, such as how this can influence the course of the infection in these animals and its transmissibility. Dogs demonstrated a high infection rate by *T. cruzi* as observed by positive serological results (92% in 2005 and 76% in 2007). The absence of positive parasitological tests confirmed their poor infectivity potential but their importance as sentinel hosts of *T. cruzi* transmission.

## Introduction

The Trypanosomatidae family is a group composed of flagellate protozoa that infect invertebrates, vertebrates, and plant hosts (Hoare, 1966; Vickerman, 1976). Although it is a well-studied group, new findings are continuously reported given that new parasite-host interactions and species/genotype diversity inside this family have been increasingly observed, especially after the advancement of molecular biology tools (d’Avila-Levy et al., 2016; Dario et al., 2017a; Maslov et al., 2019; Rangel et al., 2019; Rodrigues et al., 2019; Dario et al., 2021a).

The parasite species of this family are traditionally classified according to their developmental forms in their hosts during their life cycle (Hoare, 1966). Monoxenous trypanosomatids are those that evolved in only one host, namely, invertebrates. An example within this group is the parasite species from the genus *Crithidia*, which were first described to infect the *Anopheles maculipennis* mosquito (Léger, 1902) and are now recognized to infect bees, reduviids, and hoverflies (Langride and McGhee, 1967;  Lipa and Triggiani, 1988; Yurchenko et al., 2014; Ishemgulova et al., 2017). Monoxenic trypanosomatids were classically termed “inferior trypanosomatids”, demonstrating how much a reductionist and anthropocentric view can bypass important biological phenomena even from the sanitary point of view.

Trypanosomatid species that target an invertebrate, a vertebrate, or a plant host are named heteroxenous. The focus of this article is the *Trypanosoma* genus, which comprises species that infect vertebrates. However, the transmission cycle is only known for a minority of species, such as *Trypanosoma brucei* (sleeping sickness in humans), *Trypanosoma evansi* (surra disease in animals), and *Trypanosoma cruzi* (Chagas disease in humans) (Podlipaev, 2001). This finding reflects that the main studies are focused on human or animal parasites of economic interest, forgetting the profound interdependence between the health of all living beings and their environment.

Caatinga is a unique biome in Brazil that is found mainly in the northeast region and a small area in the southeast, namely, north of Minas Gerais state. The dominant climate is semiarid, and the term Caatinga means white forest in the indigenous language (Silva et al., 2004; Souza et al., 2015). Caatinga is the third most degraded biome in Brazil (Myers et al., 2000), and approximately 80% of the vegetation is completely modified due to extractivism and agriculture. Most of these areas are currently in the early or intermediate stages of ecological succession (Araújo Filho, 1996). As a result of these profound changes, the Caatinga presents large extensions where desertification is already noted (Souza et al., 2015). This biome, which represented an important endemic area for Chagas disease (Sousa et al., 2020) exhibits a diverse and unique fauna, comprising approximately 1307 animal species (Instituto Chico Mendes de Conservação da Biodiversidade, 2018). Caatinga harbors most of the triatomine species diversity in Brazil together with the Cerrado (Gurgel-Gonçalves et al., 2012), and *T. cruzi* infection is observed in mammals from five different orders in this biome: Artiodactyla, Carnivora, Cingulata, Didelphimorphia, and Rodentia (Jansen et al., 2018).

The order Didelphimorphia is one of the oldest and most important hosts of *Trypanosoma* species, including *T. cruzi* (Jansen et al., 2018; Rodrigues et al., 2019; Jansen et al., 2020), having as its main representative marsupials of the genus *Didelphis* (Jansen et al., 2015). The genus *Didelphis* presents a broad range distribution in nature due to its ability to adapt to different ecological niches, including anthropized environments (Olifiers et al., 2015). Therefore, it is considered a biomarker of degraded environments. These animals are nomadic, solitary, take refuge in holes and tree foliage, and are excellent climbers (Jansen et al., 2018). They are able to use all forest strata and are therefore in contact with the different transmission cycles of *T. cruzi* (Jansen et al., 2020).

The Rodentia order is the most diverse order within the small wild mammal taxa, and these animals can be found in different environments, ranging from tropical forests to deserts, plateaus to plains, and wild to urban environments. In nature, they are able to circulate through different forest strata and are found in terrestrial, arboreal, and semiaquatic areas (Wilson and Reeder, 2005). In relation to *T. cruzi* transmission, some studies have reported *T. cruzi* infection in rodents in Latin America (Herrera et al., 2005; Yeo et al., 2005; Herrera et al., 2007b; Vaz et al., 2007; Roque et al., 2008; Ramsey et al., 2012; Charles et al., 2013; Orozco et al., 2014; Herrera et al., 2015; Jansen et al., 2015; Morales et al., 2017). Several species of this order are highly susceptible to experimental infection by *T. cruzi*, demonstrating high and long-lasting parasitemia (Roque et al., 2005). However, it was suggested that this taxon plays a secondary role as a reservoir in the wild environment (Jansen et al., 2018) because the infection rate of wild rodents by *T. cruzi* is low, potentially due to the restricted use of the environment (restricted home range), which leads to less contact with other infected mammals and vectors (Jansen et al., 2015).

The role of dogs in the transmission cycle of *T. cruzi* can vary according to each region. In Brazil, dogs are highly exposed to *T. cruzi* infection based on high rates of positive serological tests. However, the detection of the parasite in blood smears or parasite isolation by hemoculture and xenodiagnosis is rare (Roque and Jansen, 2008; Noireau et al., 2009; Xavier et al., 2012, Jansen et al., 2018). A distinct scenario was described in Argentina, especially in the Gran Chaco region, where dogs are one of the main *T. cruzi* reservoirs, as demonstrated by high transmissibility competence (Gürtler et al., 2007; Gürtler and Cardinal, 2015). In Brazilian regions that present triatomines and small wild mammals with high *T. cruzi* infection rates, dogs are usually also exposed to infection as observed by serological positive tests but primarily without positive parasitological tests to date. Therefore, monitoring these animals through parasitological and serological surveys is an indication of the presence of a parasite transmission cycle occurring among wild free-ranging mammals in the area (Roque et al., 2008; Roque and Jansen, 2008; Pineda et al., 2011; Xavier et al., 2012).

Mixed infections by trypanosomatids, mainly in wild mammals, are poorly understood and rarely studied in animals. However, these infections can provide important epidemiological data because coinfections can modify the course of infection of a parasitosis. As the one-parasite-one-disease concept generally predominates, the focus has always been on *T. cruzi* or *Leishmania* sp., and the possible coinfecting trypanosomatids have been relegated to the background. Mixed infections between different trypanosomatid species and genotypes are very common in nature (Barbosa et al., 2017; Dario et al., 2017a; Jansen et al., 2018; Patiño et al., 2021). The determination of mixed infections is essential for the understanding of parasitological events, especially the resulting interactions between the parasite and its hosts. The aim of this study was to determine the trypanosomatid infection and richness in the synantropic *Rattus rattus*, *Didelphis albiventris*, and dogs in the *T. cruzi* transmission cycle in this semiarid Caatinga biome region.

## Material and Methods

### Study Area and Trypanosomatid DNA Sampling

**Figure 1:**
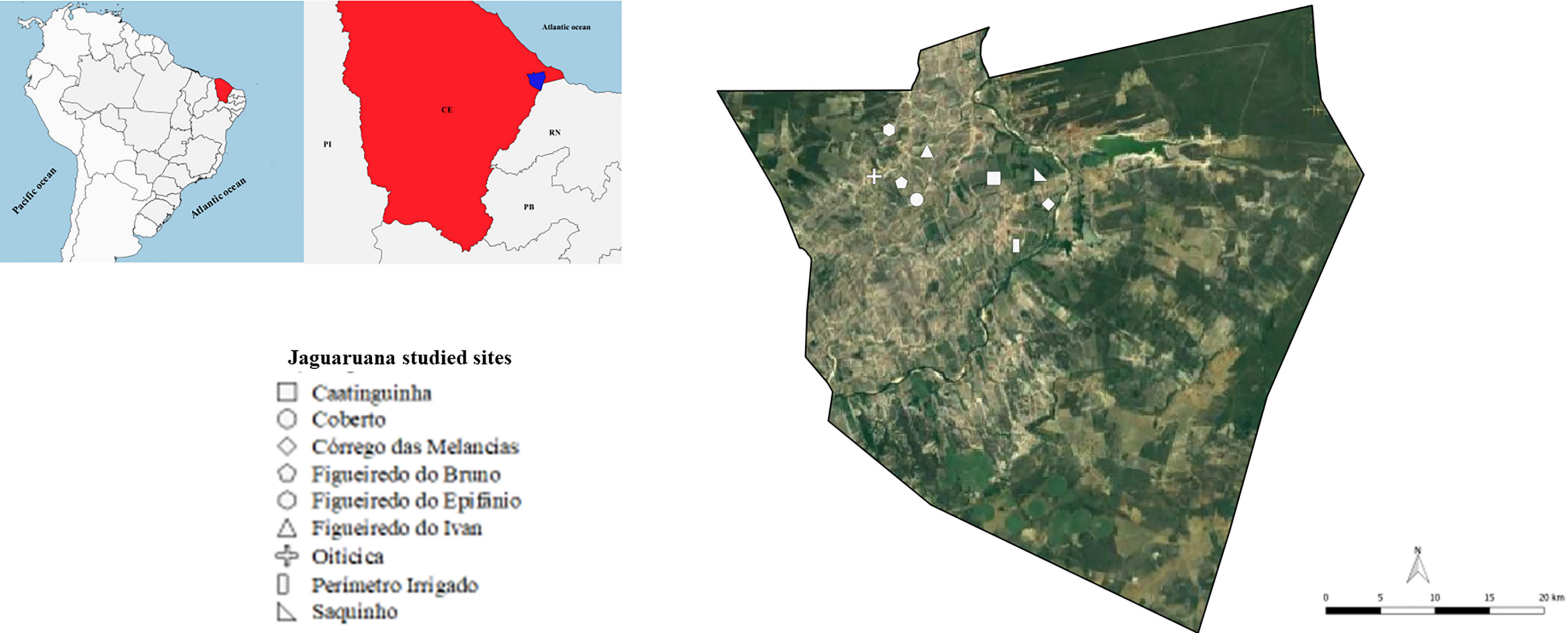
Jaguaruana municipality map, Ceará state, northeast Brazil. The red area represents Ceará state, and the blue area represents Jaguaruana municipality. The red area represents Ceará state, and the blue area represents Jaguaruana municipality. Each dot represents locations where DNA samples were obtained. CE, Ceará state; PI, Piauí state; RN, Rio Grande do Norte state; PB, Paraíba state. Data sources: Instituto Brasileiro de Geografia e Estatística – IBGE (www.ibge.gov.br); Google Earth (https://www.google.com.br/intl/pt-BR/earth/). The map was constructed using QGIS software v.2.18.15. Unfortunately, the location of the two rats included in this study could not be identified.

Jaguaruana municipality (4°50’02” S, 37°46’51” W) is in the Caatinga biome of Ceará state, northeast Brazil (**Figure 1**). According to the Brazilian Institute of Geography and Statistics (IBGE, 2010), Jaguaruana has an area of 867,251 km² and a total population of 32,239 inhabitants. DNA samples of the trypanosomatids isolated from hemocultures of *R. rattus*, *D. albiventris*, and triatomines (*Rhodnius nasutus* and *Triatoma brasiliensis*) were deposited in the DNA library of the *Trypanosoma* from wild and domestic mammals and vectors collection (COLTYP/Fiocruz). Forty-two deposited *Trypanosoma* spp. samples derived from *R. rattus*, *D. albiventris*, *R. nasutus*, and *T. brasiliensis* were submitted to DNA extraction (**Table 1**). The hemocultures were washed with phosphate-buffered saline solution, 100 µg/mL proteinase K (Invitrogen, Carlsbad, CA, USA), and 0.5% sodium dodecyl sulfate, and the samples were incubated at 56°C for 2 h. After this step, the DNA was extracted using the phenol–chloroform method (Sambrook and Russel, 2001). The trypanosomatid and DNA samples were obtained from opossums, rats, and triatomines collected in the following locations: Caatinguinha, Coberto, Córrego das Melancias, Figueiredo do Ivan, Figueiredo do Bruno, Figueiredo do Epifânio, Oiticica, Perímetro Irrigado, and Saquinho (**Figure 1**) in 2001, 2002, 2004, 2005, and 2006. These areas were considered periurban areas and were defined as transitional or surrounding regions where urban and rural activities overlapped and landscape characteristics were subject to rapid modifications due to anthropogenic activity (Lima et al., 2012).

**Table 1:** DNA sample origin and identification in Jaguaruana municipality, Ceará state, northeast Brazil.

Sample ID	Mammalian/triatomine host	Location	Year
COLTRYP0001	*Didelphis albiventris*	Figueiredo do Bruno	2005
COLTRYP0011	*Didelphis albiventris*	Caatinguinha	2005
COLTRYP0037	*Rattus rattus*	Caatinguinha	2006
COLTRYP0038	*Rattus rattus*	Caatinguinha	2006
COLTRYP0039	*Rattus rattus*	Caatinguinha	2006
COLTRYP0044	*Didelphis albiventris*	Figueiredo do Bruno	2001
COLTRYP0048	*Didelphis albiventris*	Córrego das Melancias	2004
COLTRYP0087	*Didelphis albiventris*	Caatinguinha	2005
COLTRYP00100	*Didelphis albiventris*	Caatinguinha	2004
COLTRYP00128	*Didelphis albiventris*	Caatinguinha	2004
COLTRYP00171	*Rattus rattus*	Córrego das Melancias	2004
COLTRYP00172	*Rattus rattus*	Caatinguinha	2005
COLTRYP00181	*Didelphis albiventris*	Caatinguinha	2005
COLTRYP00192	*Didelphis albiventris*	Córrego das Melancias	2005
COLTRYP00193	*Didelphis albiventris*	Figueiredo do Ivan	2005
COLTRYP00195	*Didelphis albiventris*	Caatinguinha	2005
COLTRYP00199	*Didelphis albiventris*	Caatinguinha	2004
COLTRYP00200	*Didelphis albiventris*	Córrego das Melancias	2005
COLTRYP00204	*Didelphis albiventris*	Coberto	2005
COLTRYP00207	*Rattus rattus*	Saquinho	2004
COLTRYP00239	*Didelphis albiventris*	Coberto	2005
COLTRYP00244	*Didelphis albiventris*	Figueiredo do Ivan	2005
COLTRYP00249	*Didelphis albiventris*	Caatinguinha	2005
COLTRYP00251	*Didelphis albiventris*	Caatinguinha	2005
COLTRYP00253	*Didelphis albiventris*	Córrego das Melancias	2005
COLTRYP00258	*Didelphis albiventris*	Córrego das Melancias	2004
COLTRYP00266	*Didelphis albiventris*	Figueiredo do Bruno	2005
COLTRYP00267	*Didelphis albiventris*	Córrego das Melancias	2006
COLTRYP00268	*Didelphis albiventris*	Caatinguinha	2006
COLTRYP00269	*Didelphis albiventris*	Figueiredo do Epifanio	2004
COLTRYP00285	*Didelphis albiventris*	Caatinguinha	2006
COLTRYP00287	*Rattus rattus*	Caatinguinha	2006
COLTRYP00290	*Didelphis albiventris*	Perímetro Irrigado	2006
COLTRYP00300	*Didelphis albiventris*	Caatinguinha	2006
COLTRYP00307	*Rhodnius nasutus*	Caatinguinha	2006
COLTRYP00308	*Triatoma brasiliensis*	Oiticica	2006
COLTRYP00309	*Rhodnius nasutus*	Caatinguinha	2006
COLTRYP00866	*Rattus rattus*	Caatinguinha	2004
COLTRYP00867	*Rattus rattus*	Figueiredo do Ivan	2001
COLTRYP00868	*Didelphis albiventris*	Caatinguinha	2006
Not informed	*Rattus rattus*	Not informed	2001
Not informed	*Rattus rattus*	Not informed	2004

### Trypanosomatid Species Molecular Characterization and Next-Generation Sequencing

To identify *T. cruzi* infection, the DNA samples were submitted to multiplex PCR to amplify the nontranscribed spacer of the miniexon gene (Fernandes et al., 2001) for the identification of TcI (DTU I – 200 bp), TcII (DTU II/V/VI – 250 bp), zymodeme 3 (DTU III/IV – 150 bp), and *T. rangeli* (100 bp) as well as mixed infections. All reactions included distilled water as a negative control. The following *T. cruzi* strains served as positive controls: TcI-SylvioX/10cl1; TcII-Esmeraldocl3; TcIII-M5631cl5; TcIV-92122102R; and TcV/VI-SC43cl1. One *T. rangeli* isolate (Choco) also served as a positive control. The PCR products were subjected to electrophoresis in a 2% agarose gel, which was stained with ethidium bromide solution and visualized under UV light. Restriction fragment length polymorphism (RFLP) analysis of the nuclear 1f8 gene and DNA digestion by the Alw21I enzyme were performed to distinguish DTUs TcII from TcV and TcVI  (Rozas et al., 2007). The digested products were electrophoresed in a 3% agarose gel, stained with ethidium bromide solution, and visualized under ultraviolet light.

Additionally, the samples were submitted to nested-PCR for amplification of the small subunit (SSU) rDNA gene (650 bp) (Noyes et al., 1999; Smith et al., 2008). The amplified products were subjected to electrophoresis as described for mini-exon multiplex PCR and purified using the Illustra GFX PCR DNA and gel band purification kit (GE Healthcare Life Sciences, Little Chalfont, Buckinghamshire, UK). The forward and reverse DNA strands of samples were sequenced using BigDye Terminator v3.1 Cycle Sequencing Kit (Applied Biosystems, Foster City, CA, USA) on an ABI 3730 DNA sequencer available on the PDTIS/FIOCRUZ sequencing platform.

For NGS, DNA samples were amplified using the primers S825F (5’-ACCGTTTCGGCTTTTGTTGG-3’) and S662R (5’-GACTACAATGGTCTCTAATC-3’) (Maslov et al., 1996; Barbosa et al., 2017) of the 18S rDNA gene for partial Trypanosomatidae gene sequences. These primers contained Illumina MiSeq adapter sequences. For the polymerase chain reaction (PCR), the 2X Phusion Flash High Fidelity Master Mix (Thermo Fisher, Waltham, MA, USA) was used based on the following reaction parameters: 10 µl of the master mix, 0.8 μM of each primer, and 2 to 5 µl of DNA in a final volume of 20 µl. The DNA amplification was adapted from the cycling recommended by the manufacturer as follows: an initial denaturation step at 98°C for 10 s; 30 cycles of denaturation at 98°C for 1 s, annealing at 55°C for 10 s, and extension at 72°C for 15 s; and a final extension step at 72°C for 1 min. The amplified products were subjected to electrophoresis in a 2% agarose gel containing SYBR Safe Gel Stain (Invitrogen, Waltham, MA, USA) and visualized under ultraviolet light to observe the band of interest (~350 base pairs). The amplified samples were purified (Illustra GFX PCR DNA and gel band purification kit, GE Healthcare Life Sciences, Little Chalfont, Buckinghamshire, UK) and quantified by fluorimetry (Qubit 2.0, Life Technologies, Carlsbad, CA, USA).

For the DNA library preparation, a second PCR was performed to introduce Illumina indices (Nextera XT Index Kit v2, Illumina, San Diego, CA, USA). The 2X Phusion Flash High Fidelity Master Mix (Thermo Fisher, Waltham, MA, USA) was also used, and 100 ng of each amplified product was applied. The PCR cycling in this step was as follows: an initial denaturation step at 98°C for 10 s; 8 cycles of denaturation at 98°C for 1 s, annealing at 55°C for 10 s and extension at 72°C for 15 s; and a final extension step at 72°C for 1 min. After PCR, samples were purified using magnetic beads (Ampure Beads Beckman Coulter) and quantified (Qubit 2.0, Life Technologies, Carlsbad, CA, USA). The samples were pooled to equimolar concentrations, and sequencing was performed on a MiSeq sequencer (Illumina, San Diego, CA, USA) using the MiSeq Reagent v2 kit (500 cycles) (2 x 250 paired-end reads).

### Tripanosomatid Species Delimitation and Phylogenetic Analysis

SSU rDNA analysis: To obtain the consensus sequences, each forward and reverse sample was assembled and edited using the SeqMan program (DNASTAR Lasergene). The obtained sequences were aligned and corrected using MegaX software (Kumar et al., 2018). The sequences were compared with nucleotide sequences deposited in GenBank using the BLAST (Basic Local Alignment Search Tool – https://blast.ncbi.nlm.nih.gov/Blast.cgi - algorithm for trypanosomatid species identification).

Denoised Operational Taxonomic Unit (ZOTU) NGS analysis: To obtain reads with base calling quality greater than or equal to 99.9%, the reads generated were filtered and edited using the Sickle program (Joshi and Fass, 2011). The quality-selected reads were mapped against a trypanosomatid sequence database built from 18S rDNA gene sequences (**Supplementary Material S1**) available in the SILVA v132 database project (Quast et al., 2013) using the Bowtie 2 program (Langmead and Salzberg, 2012). To obtain a consensus read, forward and reverse reads were overlapped using the Flash 1.2.11 program (Magoč and Salzberg, 2011). The reads for which the forward sequence did not overlap with the reverse sequence were submitted to the fastq_mergepairs command available at USEARCH v11 (drive5.com/usearch/). For ZOTU identification, unique reads were identified using the command dereplication in VSEARCH 2.17.1 (Rognes et al., 2016). The UNOISED (Edgar and Flyvbjerg, 2015; Edgar 2016) algorithm was used to cluster the correct biological sequences in the reads, and an OTU table was constructed using USEARCH v11 (drive5.com/usearch/). The identification of trypanosomatid species and their genotypes was initially determined using the Basic Local Alignment Search Tool (BLAST) algorithm available on the National Center for Biotechnology Information (NCBI) website. As a read cutoff for determining the species/genotype occurrence per sample, the total reads per sample obtained in the ZOTU table were normalized to 100,000 reads, and ZOTUs that presented ≤ 150 reads in the sample were excluded from the analysis.

Amplicon Sequence Variant NGS analysis: The NGS-generated data were imported into the R v3.6.2 environment, wherein all the analyses were performed (R Development Core, 2021). Sequences were analyzed using the DADA2 package v1.14.0 following the analysis pipeline as given in the tutorial (https://benjjneb.github.io/dada2/tutorial.html) (Callahan et al., 2016). Furthermore, the same sequence database used for the ZOTU analysis was used in the ASV analysis (**Supplementary Material S1**). The ASV table and assigned taxonomy and sample metadata information were combined as a phyloseq object (phyloseq package version 1.30.0) (McMurdie & Holmes, 2013).

The ZOTU and ASV reads were aligned to other trypanosomatid 18S rDNA retrieved from the GenBank database using the L-INS-i algorithm in MAFFT v.7.0 (Katoh, 2013). The alignment was visualized and manually edited on MegaX (Kumar et al., 2018). Maximum likelihood (ML) estimation and Bayesian inference (BI) were performed for trypanosomatid species/genotype identification and genetic clustering. For each phylogenetic analysis, the best base substitution models were chosen according to the corrected Akaike information criterion (cAIC) in ModelFinder (Kalyaanamoorthy et al., 2017). ML reconstruction was performed in the IQ-Tree program (Nguyen et al., 2015) on PhyloSuite v.1.2.2 (Zhang et al., 2020). Ultrafast bootstrapping (Hoang et al., 2018) was performed with 5000 replicates with 1000 maximum interactions and 0.99 minimum correlation coefficients for branch support. To validate this result, the SH-aLRT branch test with 5000 replicates was also applied. Bayesian tree reconstruction was performed in Bayesian Evolutionary Analysis Sampling Trees (BEAST) v2.6.2 (Bouckaert et al., 2019) using the Bayesian Markov chain Monte Carlo (MCMC) method to assign trypanosomatid species/genotypes prior to information. The birth-death model specification was used in genotype tree reconstruction, and the Yule model specification was used for species tree reconstruction. Three independent runs were performed for 20 M with sampling every 2000 generations. The runs converged and the effective sample size (ESS) were calculated after 25% of each run was excluded (burn-in) from each run in TRACER v.1.6 (Rambaut et al., 2018). Parameters greater than 500 were considered appropriate. The final tree was generated with maximum clade credibility (MCC) based on 16878 trees (burn-in = 5625) and a 0.6 posterior probability limit (PP) in Tree Annotator. The ML and BI reconstruction trees were visualized in Figtree v.1.4.3.

In addition, haplotype networks based on *T. cruzi* DTU TcI samples were generated in Network software version 5.0.1.1 (fluxus-engineering.com) to define evolutionary relationships and to observe the intraspecificity among these DTUs. The network was built using median-joining (Bandelt et al., 1999) and maximum parsimony (Polzin and Daneschmand, 2003) postprocessed clean-up procedures.

### Statistical Analysis of *Trypanosoma cruzi* DTU TcI Haplotype Diversity

*Trypanosoma cruzi* DTU TcI haplotype diversity was calculated using the Shannon, Simpson, evenness, and beta-diversity indices per location and per mammalian host. All statistical analyses were performed in R version 4.0.3 (vegan and betapart packages).

### Detection of *Trypanosoma* spp. Infection in Dogs

DNA samples were obtained from wild mammals and dogs in the surrounding areas and were investigated for *T. cruzi* infection using parasitological and serological methods. Blood samples were collected using Vacutainer^®^ tubes containing EDTA by puncturing the dog’s brachial vein with the informed consent of their owners. A questionnaire was used to record the name, age, sex, size, primary function (hunting, companionship, or protection), and anatomical peculiarities. The dogs were investigated in 2004 and 2005 in a previous study (Lima et al., 2012), and another investigation was performed in 2007 by our group.

Blood (0.3–0.6 µL) was seeded in two tubes containing Novy-Mc Neal-Nicole medium (NNN) overlaid with 2 mL liver infusion tryptose medium (LIT). Fresh blood smears were examined by optical microscopy. The remaining blood was centrifuged, and the serum was stored at −20°C. Hemocultures were examined every 2 weeks for 5 months, and the positive samples were amplified for molecular characterization and cryopreservation. The amplified samples were deposited in the Coleção de *Trypanosoma* de Mamíferos Silvestres, Domésticos e Vetores (COLTRYP/Fiocruz).

A serological survey for the detection of anti-*T. cruzi* antibodies was performed using an indirect immunofluorescence antibody test (IFAT) to evaluate infection in wild mammals and dogs (Camargo, 1966). The sera were tested with anti-dog IgG conjugate (FICT, Sigma, St. Louis, MO, USA), and a cutoff value of 1:40 was adopted. To avoid misinterpretation due to cross reactions and detect mixed infections, the dogs were also screened for *Leishmania* infection using an IFI-Leishmania Bio-Manguinhos Kit (Fiocruz). Dogs with 2x higher serologic titers for *Leishmania* than *T. cruzi* were not considered infected by *T. cruzi*. When both titers were similar and greater than the cutoff, mixed infection by both parasites was considered (Roque et al., 2008).

## Results

### Trypanosomatid Molecular Characterization of Isolates Derived From Rats, Opossums and Triatomines

Rats and opossums were demonstrated to maintain an impressive *T. cruzi* diversity, in addition to *T. rangeli* infection (**Table 2**). The results from 42 DNA samples that were submitted to mini-exon multiplex PCR (**Table 2**) showed that the majority (31) were *T. cruzi* TcI samples (26 *D. albiventris* and nine *R. rattus*). Moreover, one *D. albiventris* sample presented mixed infection by TcI and *T. rangeli*. Three samples derived from one *D. albiventris* specimen and two *R. rattus* specimens were identified as *T. cruzi* DTU TcII. One *R*. *nasutus* and the *T. brasiliensis* specimens presented infection by *T. cruzi* DTU TcI. Moreover, mixed infection by *T. cruzi* DTU TcI and *T. rangeli* was reported in the other *R. nasutus* specimen. SSU rDNA nested-PCR was performed in 42 DNA samples from rats (n=11), opossums (n=28), and triatomines (n=3). The results yielded electropherograms presenting double peaks, making it impossible to create consensus sequences and indicating mixed infections. Thirty-nine DNA samples (27 samples from *D. albiventris*, 11 from *R. rattus*, two from *R. nasutus*, and one from *T. brasiliensis*) were submitted to NGS for trypanosomatid species identification.

**Table 2:** Trypanosomatid molecular characterization of *Rattus rattus*, *Didelphis albiventris*, and triatomines by mini-exon multiplex-PCR, 18S rDNA Sanger sequencing, and next-generation sequencing.

Sample ID	Mammalian/triatomine	Mini-exon multiplex-PCR	18S rDNA sanger sequencing	ZOTU identification	ASV identification
COLTRYP0001	*Didelphis albiventris*	TcI	*T. rangeli* A^a^	Not performed	Not performed
COLTRYP0011	*Didelphis albiventris*	TcI	No consensus	TcI ZOTU1	TcI ASV1
COLTRYP0037	*Rattus rattus*	TcI	No consensus	TcI ZOTU1, TcI ZOTU2, TcI ZOTU10 merged	TcI ASV1, *T. rangeli* A and B
COLTRYP0038	*Rattus rattus*	TcI	No consensus	TcI ZOTU1, TcI ZOTU2, TcI ZOTU5, TcI ZOTU10 merged	TcI ASV1, *T. dionisii*, TcII
COLTRYP0039	*Rattus rattus*	TcI	No consensus	TcI ZOTU1, TcI ZOTU2, TcI ZOTU5, TcI ZOTU10 merged	TcI ASV1, TcIV
COLTRYP0044	*Didelphis albiventris*	TcI	No consensus	TcI ZOTU1	TcI ASV1, *T. dionisii*,
COLTRYP0048	*Didelphis albiventris*	TcI	No consensus	TcI ZOTU1, TcI ZOTU2, TcI ZOTU10 merged, TcII	TcI ASV1, TcI ASV2, TcII, *T. dionisii*
COLTRYP0087	*Didelphis albiventris*	TcI	No consensus	TcI ZOTU1, TcI ZOTU2	TcI ASV1
COLTRYP00100	*Didelphis albiventris*	TcI	No consensus	TcI ZOTU1, TcI ZOTU2, TcI ZOTU10 merged	TcI ASV2
COLTRYP00128	*Didelphis albiventris*	TcI	No consensus	TcI ZOTU1, TcI ZOTU2, TcI ZOTU10 merged	TcI ASV1
COLTRYP00171	*Rattus rattus*	TcI	No consensus	TcI ZOTU1	TcI ASV1
COLTRYP00172	*Rattus rattus*	TcI	No consensus	Tc I ZOTU1, TcI ZOTU2, TcI ZOTU5, TcI ZOTU10 merged	TcI ASV2, TcI ASV5, TcII, *T. lainsoni*
COLTRYP00181	*Didelphis albiventris*	TcI	No consensus	TcI ZOTU1	TcI ASV1, TcII, TcIV, *T. lainsoni*
COLTRYP00192	*Didelphis albiventris*	TcI	No consensus	TcI ZOTU1	TcI ASV1
COLTRYP00193	*Didelphis albiventris*	TcI	No consensus	TcI ZOTU1	TcI ASV1
COLTRYP00195	*Didelphis albiventris*	TcI	No consensus	TcI ZOTU1	TcI ASV1, *T. rangeli* A and B
COLTRYP00199	*Didelphis albiventris*	TcI	No consensus	TcI ZOTU1, TcI ZOTU2	TcI ASV2
COLTRYP00200	*Didelphis albiventris*	TcI	No consensus	TcI ZOTU1, TcI ZOTU2, TcI ZOTU10 merged	TcI ASV1
COLTRYP00204	*Didelphis albiventris*	TcI	No consensus	TcI ZOTU1, TcI ZOTU2, TcI ZOTU10 merged	TcI ASV1, TcIV
COLTRYP00207	*Rattus rattus*	TcI	No consensus	TcI ZOTU1, TcI ZOTU2, *T. rangeli* B, *Trypanosoma* sp.	TcI ASV2, *T. rangeli* B, *C. mellificae*
COLTRYP00239	*Didelphis albiventris*	TcII	No consensus	TcI ZOTU1, TcI ZOTU2, TcI ZOTU10 merged, TcII	TcI ASV2, TcII, *T. dionisii*
COLTRYP00244	*Didelphis albiventris*	TcI	No consensus	TcI ZOTU1, TcI ZOTU2, TcI ZOTU10 merged	TcI ASV1
COLTRYP00249	*Didelphis albiventris*	TcI	No consensus	TcI ZOTU1	TcI ASV1
COLTRYP00251	*Didelphis albiventris*	TcI	No consensus	TcI ZOTU1	TcI ASV1
COLTRYP00253	*Didelphis albiventris*	TcI	No consensus	TcI ZOTU1	TcI ASV1, TcII, *T. lainsoni*
COLTRYP00258	*Didelphis albiventris*	TcI	No consensus	TcI ZOTU1, TcI ZOTU2, TcI ZOTU10 merged	TcI ASV1, TcI ASV, TcI ASV13, TcI ASV3
COLTRYP00266	*Didelphis albiventris*	TcI-*T. rangeli*	No consensus	TcI ZOTU1, *T. rangeli* A, *Trypanosoma* sp.	TcI ASV1, *T. rangeli* A
COLTRYP00267	*Didelphis albiventris*	TcI	No consensus	Not amplified	Not amplified
COLTRYP00268	*Didelphis albiventris*	TcI	No consensus	TcI ZOTU1, TcI ZOTU2, TcI ZOTU10 merged	TcI ASV1, TcIV
COLTRYP00269	*Didelphis albiventris*	TcI	No consensus	TcI ZOTU1, TcI ZOTU2, TcI ZOTU10 merged	TcI ASV1, TcI ASV, TcI ASV13
COLTRYP00285	*Didelphis albiventris*	TcI	No consensus	Not amplified	Not amplified
COLTRYP00287	*Rattus rattus*	TcI	No consensus	TcI ZOTU1, TcI ZOTU2, TcI ZOTU5, TcI ZOTU10 merged, *C. mellificae*	TcI ASV1, TcII, *C. mellificae*, *T. dionisii*
COLTRYP00290	*Didelphis albiventris*	TcI	No consensus	TcI ZOTU1, TcI ZOTU2	TcI ASV1
COLTRYP00300	*Didelphis albiventris*	TcI	No consensus	Not amplified	Not amplified
COLTRYP00307	*Rhodnius nasutus*	TcI-*T. rangeli*	No consensus	TcI ZOTU1, TcI ZOTU2, TcI ZOTU5, TcI ZOTU10 merged	TcI ASV2, TcI ASV5
COLTRYP00308	*Triatoma brasiliensis*	TcI	No consensus	TcI ZOTU1, TcI ZOTU2, TcIV	TcI ASV1, TcIV
COLTRYP00309	*Rhodnius nasutus*	TcI	No consensus	TcI ZOTU1, TcI ZOTU2, TcI ZOTU5, TcI ZOTU10 merged	TcI ASV2, TcI ASV5
COLTRYP00866	*Rattus rattus*	TcII	No consensus	Not amplified	Not amplified
COLTRYP00867	*Rattus rattus*	TcI	No consensus	TcI ZOTU1	TcI ASV1, TcIV
COLTRYP00868	*Didelphis albiventris*	TcI	No consensus	TcI ZOTU1, TcI ZOTU2, TcI ZOTU5, TcI ZOTU10 merged	TcI ASV1, TcI ASV, TcI ASV13, TcI ASV3
Not informed	*Rattus rattus*	TcI	Not performed	Not performed	Not perfomed
Not informed	*Rattus rattus*	TcII	Not performed	Not performed	Not performed

a Sequence published in Dario et al., 2021b.

**Figure 2:**
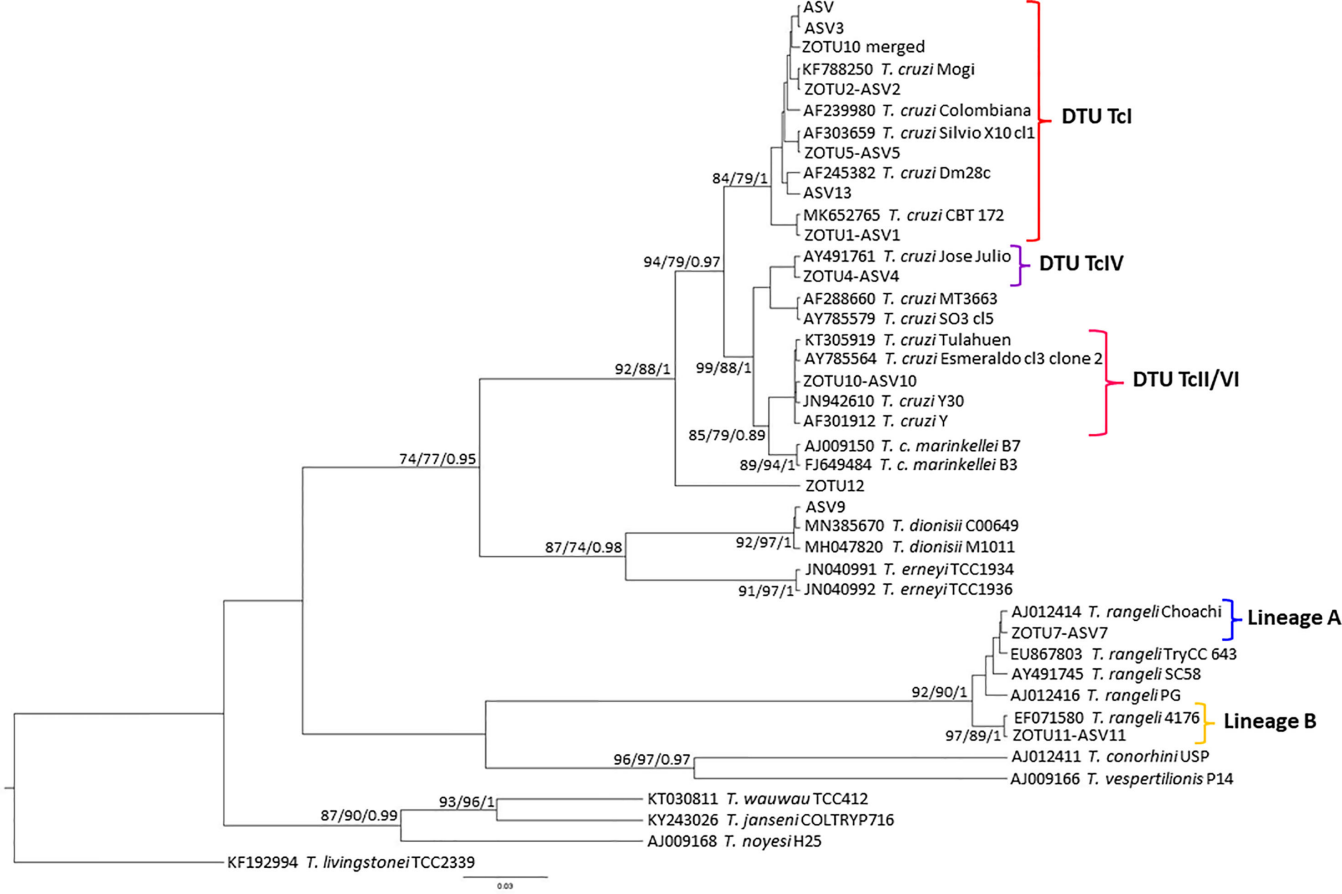
*Trypanosoma cruzi* clade phylogenetic tree based on 465bp 18S rDNA fragment length from *D. albiventris*, *R. rattus*, *R. nasutus*, and *T. brasiliensis* culture samples. The tree was inferred with TPM3uf plus gamma distribution among sites (TPM3uf+G) for ML and BI. The number at nodes corresponds to ML (ultrabootstrap and SH-aLRT) and BI (posterior probability). The scale bar shows the number of nucleotide substitutions per site. The red bracket indicates the group formed by *T. cruzi* DTU TcI; the lilac bracket indicates the sequences grouped as *T. cruzi* DTU TcIV; the pink bracket indicates the sequences identified as *T. cruzi* DTU TcII; the blue bracket indicates the sequences identified as *T. rangeli* lineage A; and the yellow bracket indicates the sequences identified as *T. rangeli* lineage B.

**Figure 3:**
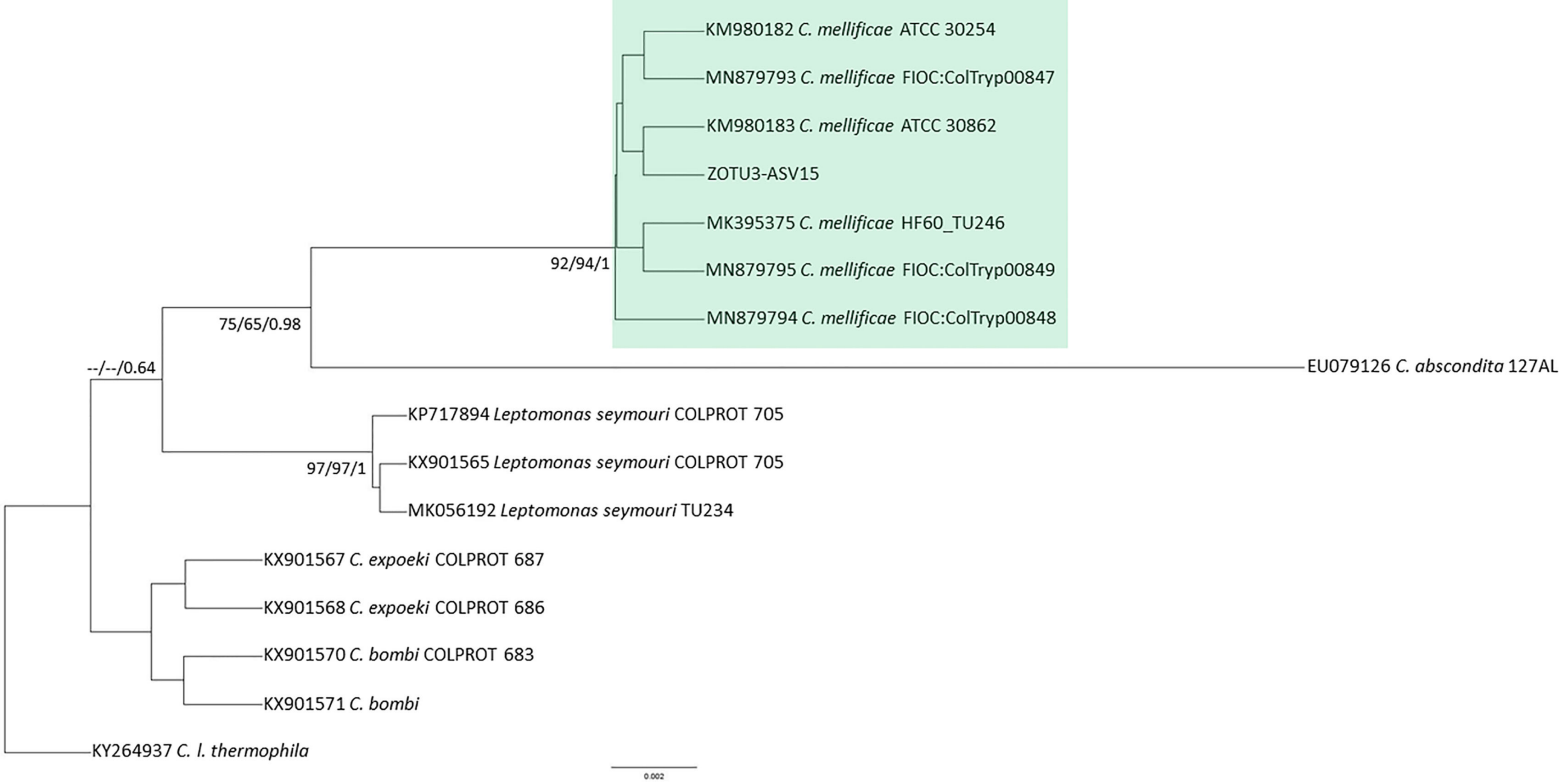
*Crithidia mellificae* phylogenetic tree based on 333bp 18S rDNA fragment length from *R. rattus* hemoculture sample. The tree was inferred with Bayesian using Tamura-Nei with equal frequencies (TrNef) model for ML and BI. The number at nodes corresponds to ML (ultrabootstrap and SH-aLRT) and BI (posterior probability). The scale bar shows the number of nucleotide substitutions per site. The green square indicates the group formed by *C. mellificae* sequences from different hosts.

Of the 35 samples (24 from *D. albiventris*, eight from *R. rattus*, two from *R. nasutus*, and one from *T. brasiliensis*) that were sequenced (**Table 2**), 10 ZOTUs were assigned to *T. cruzi* DTU TcI (ZOTU1, ZOTU2, ZOTU5, ZOTU10 merged); DTU TcII (ZOTU 10); DTU TcIV (ZOTU4); *T. rangeli* lineages A and B included ZOTU7 and ZOTU11, respectively; *Trypanosoma* sp. (ZOTU12) and *C. mellificae* (ZOTU3) (**Figures 2** and **3**). Eleven samples (31, 43%) presented single infection exclusively by *T. cruzi* TcI ZOTU1, and 24 samples (68.57%) presented mixed infection from two to five ZOTUS. ZOTU1 was predominant and was present in all samples. The DTU TcI was found in all mammalian hosts and vectors. DTU TcII was found in *D. albiventris*, and DTU TcIV was found in *T. brasiliensis* (**Table 2**). The sequence identified as *Trypanosoma* sp. (ZOTU12) found in mammals grouped together with sequences from the subgenus *Schizotrypanum* species (*T. cruzi*, *T. c. marinkellei*, *T. dionisii*, and *T. erneyi*) in a more basal branch (**Figure 2**). *Crithidia mellificae* was identified in two *R*. *rattus* specimen (**Table 2**). *Trypanosoma rangeli* was detected in both *R. rattus* and *D. albiventris* (**Table 2**).

**Figure 4:**
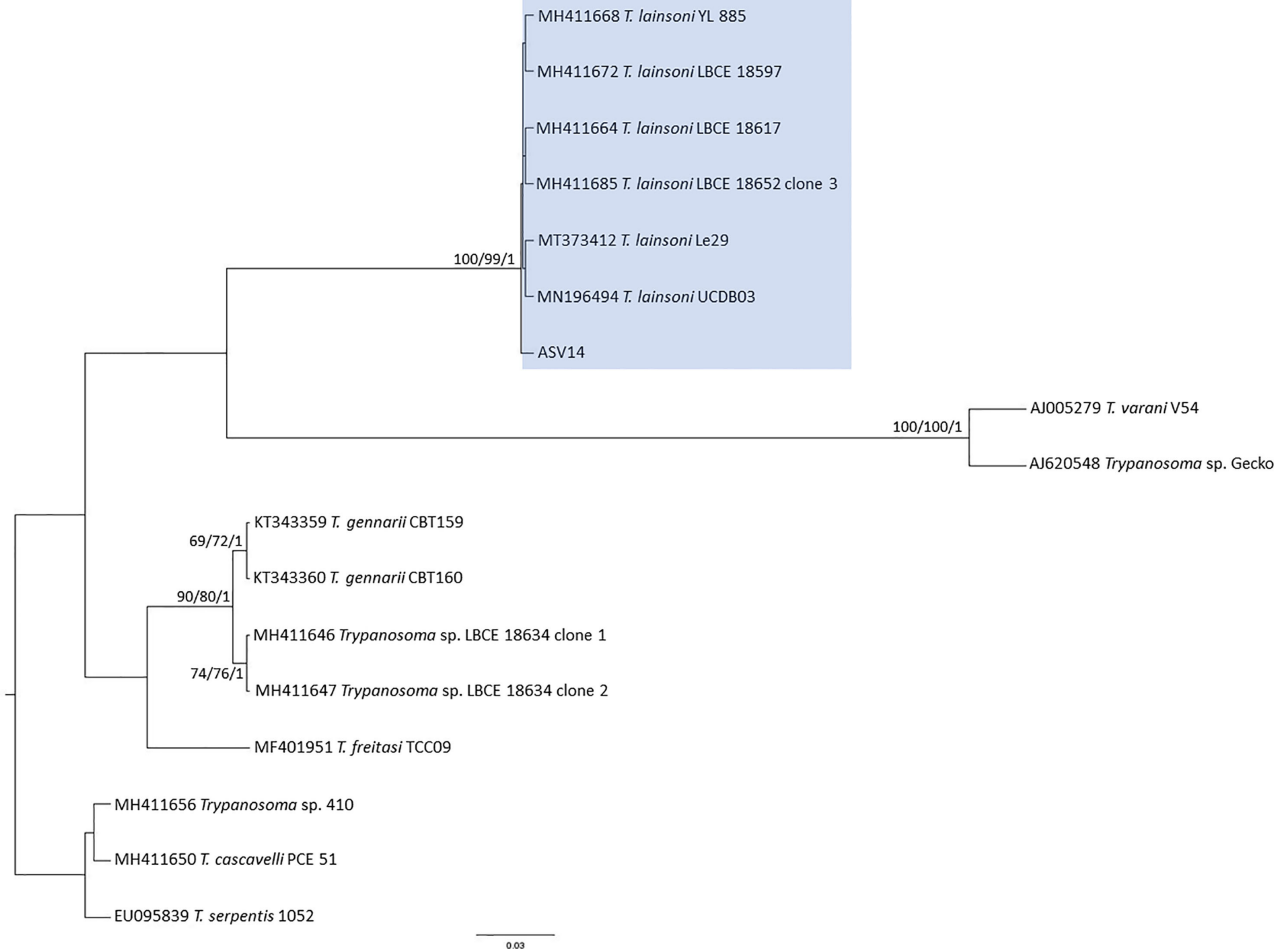
*Trypanosoma lainsoni* phylogenetic tree based on 492bp 18S rDNA fragment length from *D. albiventris* and *R. rattus* hemoculture samples. The tree was inferred with Bayesian using transition model with plus gamma distribution among sites (TIM3+G) model for ML and BI. The number at nodes corresponds to ML (ultrabootstrap and SH-aLRT) and BI (posterior probability). The scale bar shows the number of nucleotide substitutions per site.

Thirteen ASVs were assigned to *T. cruzi* DTU TcI (ASV, ASV1, ASV2, ASV3, ASV5, ASV13), DTU TcII (ASV10), DTU TcIV (ASV4), *T. rangeli* lineages A (ASV7) and B (ASV11), *T. dionisii* (ASV9) (**Figure 2**), *T. lainsoni* (ASV14) (**Figure 4**) and *C. mellificae* (ASV15) (**Figure 3**). Thirteen samples (37, 14%) presented a single infection by *T. cruzi* TcI ASV1 (n=11) and TcI ASV2 (n=2), and 22 samples (62.85%) presented a mixed infection from two to four ASVs (**Table 2**). ASV1 was predominant, but seven samples (**Table 2**) of this amplicon sequence variant were not identified. Both *D. albiventris* and *R. rattus* presented infection by DTUs TcII and TcIV, *T. rangeli* A and B, *C. mellificae*, *T. dionisii*, and *T. lainsoni* (**Table 2**).

**Figure 5:**
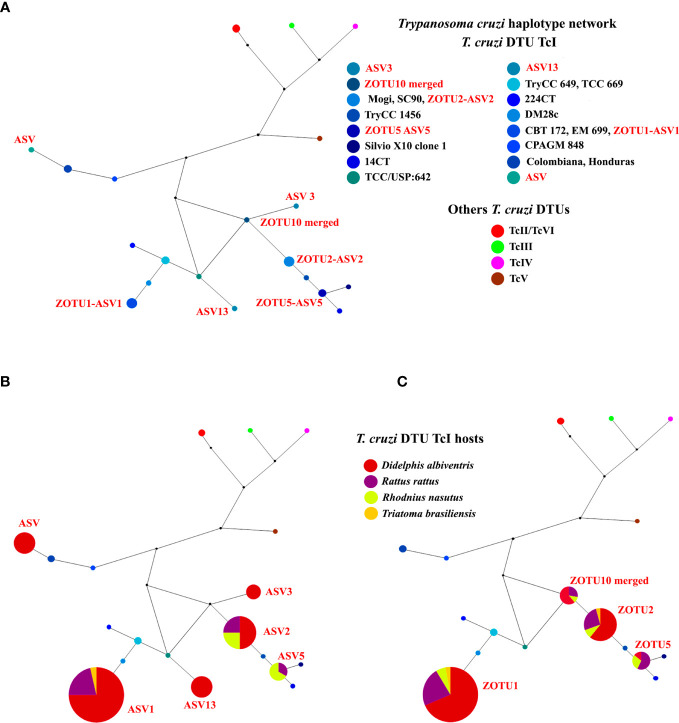
*Trypanosoma cruzi* DTU TcI haplotype network from *D. albiventris*, *R. rattus*, *R. nasutus* and *T. brasiliensis* culture samples. Networks were constructed with 18S rDNA **(A)** The size of each blue node is proportional to the haplotype frequency. **(B)** ASV haplotype per mammal and triatomine hosts. **(C)** ZOTU haplotype distribution per mammal and triatomine hosts. The small black circle represents the median vector, which can be interpreted as an unsampled sequence or an extinct ancestral sequence.

We also observed a *T. cruzi* DTU TcI intraspecific diversity. According to the haplotype network, four ZOTU and six ASV TcI haplotypes were observed to infect *D. albiventris*, *R. rattus*, *R. nasutus*, and *T. brasiliensis* (**Figure 5**). In the analysis, we observed that three ZOTUs (ZOTU1, ZOTU2, and ZOTU5) and ASVs (ASV1, ASV2, and ASV5) were the same haplotypes. We also observed five new TcI haplotypes: ZOTU5-ASV5, ZOTU10 merged, ASV, ASV3, and ASV13 (**Figure 5**). ASV, ASV3, and ASV13 were exclusively detected in *D. albiventris* (**Figure 5B**).

**Figure 6:**
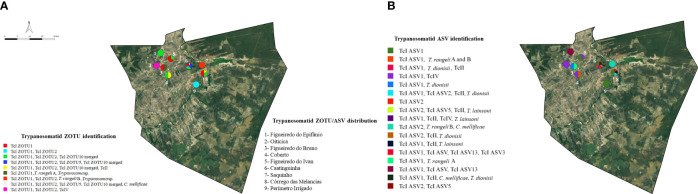
Trypanosomatid distribution map in opossum, rats, and triatomines in Jaguaruana municipality, Ceará state, Brazil. Each color represents a ZOTU **(A)** and ASV **(B)** identified. According to the ZOTU map **(A)**, the Caatinguinha location exhibited the greatest occurrence of *T. cruzi* DTU TcI; Figueiredo do Bruno and Saquinho presented greater *Trypanosoma* spp. diversity. In the ASV map **(B)**, Caatinguinha and Córrego das Melancias presented the greatest trypanosomatid diversity. Data sources: Instituto Brasileiro de Geografia e Estatística – IBGE (www.ibge.gov.br); Google Earth (https://www.google.com.br/intl/pt-BR/earth/). The map was constructed using QGIS software v.2.18.15.

According to the ZOTU-ASV distribution map (**Figure 6**), *T. cruzi* DTU TcI occurs in all the locations studied. In the ZOTU distribution, Caatinguinha presented the greatest diversity of *T. cruzi* DTU TcI, where the four haplotypes were observed and *C. mellificae* was identified. *Trypanosoma rangeli* and *Trypanosoma* sp. occurred exclusively in Saquinho and Figueiredo do Bruno; Córrego das Melancias, Coberto and Oiticica were the locations where DTU TcII and TcIV were identified, respectively (**Figure 6A**). Caatinginha presented the greatest trypanosomatid species distribution according to the ASV map followed by Córrego das Melancias (**Figure 6B**).

### Diversity Statistical Analysis of *Trypanosoma cruzi* TcI

For the ZOTU analysis of the *T. cruzi* DTU TcI, the Caatinguinha location showed the greatest diversity of *T. cruzi* TcI haplotypes. *Rattus rattus* and *D. albiventris* TcI infection displayed the same diversity – the four DTU TcI haplotype occurrence, however, in *R. rattus*, TcI presented a greater distribution in mixed infection. In the ASV, *D. albiventris* presented a greater TcI distribution, and *R. rattus* presented a greater trypanosomatid diversity. The Caatinguinha location presented the greatest trypanosomatid diversity, followed by Córrego das Melancias.

### *Trypanosoma cruzi* Parasitological and Serological Survey in Dogs

The dogs examined in 2004 and 2005 as well as those examined in 2007 (n= 195) at Matinho, Dió, Saquinho, Córrego das Melancias, Figueiredo do Bruno, Figueiredo do Ivan, Caatinguinha, and Perímetro Irrigado displayed one common trait: none of the animals presented positive results for fresh blood and hemoculture exams. In total, 86/195 (44.1%) of the dogs presented serological titers for *T. cruzi* (**Table 3**). Analyzing the results per year and locations (**Table 3**), 2005 and 2007 presented higher *T. cruzi* infection rates in dogs.

**Table 3:** *Trypanosoma cruzi* infection of dogs demonstrated by serological exams from Jaguaruana municipality, Ceará state, Brazil.

Year	2004	Positive *T. cruzi* infection	2005	Positive *T. cruzi* infection	2007	Positive *T. cruzi* infection
**Location**	Matinho	0/22	Córrego das Melancias	4/4	Caatinguinha	14/26
	Dió	0/28	Figueiredo do Bruno	2/3	Córrego das Melancias	19/19
	Saquinho	0/35	Dogs with no location information	4/5	Perímetro Irrigado	19/23
			Figueiredo do Ivan	2/2	Dió	21/28
**Total**		**0/85**		**13/14 (92.8%)**		**73/96 (76%)**

## Discussion

In this study, three findings deserve attention: 1) the richness of *Trypanosoma* spp. found in *D. albiventris* but also in *R. rattus* in a rural area, therefore not representative of the original Caatinga landscape; 2) the first finding of *C. mellificae* infection in *R. rattus*; and 3) the richness of uncharacterized genotypes of *T. cruzi*. The aim of this study was to characterize trypanosomatid richness in *D. albiventris* and *R. rattus* and *T. cruzi* infection in dogs from a locality of the Caatinga biome that years ago was endemic for Chagas disease. Currently, no cases of children under 10 years of age have been reported (Lima et al., 2012 and unpublished results 2007), showing the results of the successful South cone initiative along with popular awareness of the disease and a certain improvement in residences.

We used the mini-exon target as a first attempt to characterize the isolates from *D. albiventris* and *R. rattus*. This technique showed that most isolates were infected by *T. cruzi* DTU TcI in a single infection except for one isolate that presented mixed infection with *T. rangeli*. Three isolates of *R. rattus* were demonstrated to be TcII by RFLP-PCR. The mini-exon target is a good molecular target to characterize *T. cruzi* DTU TcI. However, as we deal with sylvatic environment samples, which often present mixed infections, the diagnosis of the infection is unclear. Therefore, we tried to characterize the samples using SSU rDNA through Sanger sequencing, which also failed because the samples presented a double peak in the electropherogram, confirming the probably mixed infections. This is a limitation of the methodology given that it is only possible to identify them by combining Sanger sequencing with the molecular cloning methodology for the diagnosis of mixed infection, which makes the procedure considerably more laborious (Zepeda Mendoza et al., 2015; Huggins et al., 2019).

Hence, we performed NGS, which allowed us to detect 15 trypanosomatid species/genotypes infecting *R. rattus*, *D. albiventris*, and triatomines in single and mixed infections. NGS allowed the identification of a broad range of samples simultaneously, in addition to identifying the possible parasite species circulating in a given sample, even using culture isolate samples, which have gone through parasite species selection process, which could affect the results observed. Some studies have already used this methodology to identify trypanosomatids in samples from different mammal species, and it was possible to identify even more than three species in a single sample and even species of the Kinetoplastea class infecting mammals (Barbosa et al., 2017; Dario et al., 2017a; Dario et al., 2017b; Cooper et al., 2018; Huggins et al., 2019; Patiño et al., 2021). This is a promising methodology in parasitological studies that is able to unravel a world that we did not have access to before due to diagnostic limitations. This method will offer new knowledge, such as new species and genotypes and their phylogenetical relations. Here, we observed a trypanosomatid sequence that phylogenetically grouped together with sequences from the *T. cruzi* clade as well as a previously undescribed host-parasite interaction, namely, *R. rattus* infected by *C. mellificae*. In addition, according to Austen and Barbosa (2021), mixed infection detection in a host and the understanding of multihost parasite dynamics are priorities from now on, and detection of this type of infection is only possible through NGS.

*Rattus rattus* is a synanthropic species that frequents human living areas. They are considered to be of economic and sanitary importance, as they cause damage to food stocks and are associated with the transmission of several zoonoses that are very general in their diet, and they present a great climbing ability. Rodents are usually suggested to play a secondary role as reservoirs of *T. cruzi* in the sylvatic environment (Rademaker et al., 2009; Jansen et al., 2018). However, this notion was not observed in this Caatinga region. Animals from this order can be involved in the *T. cruzi* transmission cycle in different ways: a) rodents can share some of their microhabitats with triatomines of the genera *Triatoma* and *Panstrongylus* (Carcavallo et al., 1998); b) as they are one of the main predation targets, rodents are important in the study of the transmission cycle of *T. cruzi*; and c) some rodent species are used in the peridomestic environment and thus may act as links between the transmission cycles (Roque et al., 2008). This work demonstrates a richness of trypanosomatids that was completely unknown in *R. rattus* and raises the question - how are these parasites that are in such low parasitemias transmitted?

*Didelphis albiventris*, similar to the other species of the genus, is considered a sylvatic taxon characterized by its high competence in adapting to artificial habitats. In fact, opossums are extremely adaptable to both the environment and diet. In the forest, opossums are also skilled tree climbers, although they are somewhat clumsy on the ground. They prey on eggs and small vertebrates as well as insects. The interaction of opossums with humans is quite old. These animals use human buildings as shelters, whereas humans often use opossums as a source of protein. Marsupials from the *Didelphis* genus are the main reservoir of *T. cruzi* in different biomes, as demonstrated by high rates of positive hemocultures (Jansen et al., 2018). These animals present generalist habits, use the different forest strata and easily adapt to modified environments, especially the peridomestic environment (Jansen et al., 2018). This set of events explains why these animals always have high rates of infection by *T. cruzi*, and this would not be different in the Caatinga.

According to our results, the *T. cruzi* transmission cycle has different profiles in nature, and the animals involved in transmission in this region of the Caatinga biome include *D. albiventris* and *R. rattus* species. Both studied species, but mainly *Didelphis* spp., are considered important reservoirs of *T. cruzi* (Jansen et al., 2015; Jansen et al., 2018). Moreover, in the studied localities, we observed these mammal species display subpatent parasitemias by an impressive richness by *T. cruzi* genotypes in the absence of new human cases, showing how powerful the adoption of easy prophylactic measures, such as improving the residences, may be effective against Chagas disease.

The DTUs TcI, TcII, and TcIV were previously described as circulating in small wild mammals with the latter noted in triatomines in the Caatinga biome. DTU TcI is the most common genotype found in *T. cruzi* (Zingales et al., 2012; Jansen et al., 2015; Brenière et al., 2016). DTU TcII, which was formerly associated with human disease, was the second most frequent in wild mammals, and similar findings were noted in the studied locality (Lisboa et al., 2015; Dario et al., 2017b). This is the first report of the DTU TcIV infecting triatomine in the Caatinga biome. This finding, which was previously observed only in triatomines of the Atlantic Forest (Dario et al., 2016; Dario et al., 2017b; Dario et al., 2018), demonstrates that this DTU can infect a variety of triatomine species and landscapes. This finding shows that no triatomine species is correlated with a certain DTU.

The DTU TcI is a genotype that presents an intraspecific genetic diversity diagnosed by several molecular markers (Salazar et al., 2006; Herrera et al., 2007a; O’Connor et al., 2007; Spotorno et al., 2008; Falla et al., 2009; Ramirez et al., 2011; Roman et al., 2018). Several subdivisions of TcI have been proposed. In Colombia, this genotype is subdivided into two groups through nuclear targets and multilocus sequence typing: one associated with wild transmission (TcI_SILV_) and the other with domestic transmission cycles (TcI_DOM_) (Ramirez et al., 2011). Here, through NGS, we were able to detect this intraspecific diversity reported in TcI, as we have observed seven different haplotypes circulating in small wild mammals and triatomines, including five new haplotypes that seem to be observed exclusively in the Caatinga biome. Roman et al. (2018) reported a set of TcI isolates from the Caatinga biome that grouped together with no other sequence from a different biome, which could indicate a geographical association. To this end, we should use the same methodology to affirm this association.

We observed different trypanosomatid distributions in Jaguaruana municipality. The Caatinguinha location was the area with the greatest diversity of TcI haplotypes and can be considered a hotspot area for this genotype. The Saquinho, Figueiredo do Bruno, and Córrego das Melancias locations presented the greatest trypanosomatid richness, and some *T. cruzi* DTUs were reported only in specific locations. Even though we are dealing with a single biome, we can affirm that microhabitats may influence parasite occurrence and diversity as observed in this region.

This is the second study employing NGS involving mammals and trypanosomatid infection in Brazil. The first was performed in the Atlantic Forest and investigated trypanosomatid infection in bats (Dario et al., 2017a). Here, we observed trypanosomatid species richness circulating in *R. rattus* and *D. albiventris*, i.e., nonflying mammals. Both studies revealed an unsusceptible richness of trypanosomatids in these distinct taxa, demonstrating that this diversity is common. Our findings reinforce the marsupial as a bioaccumulator of DTU TcI and demonstrate *R. rattus* as an important host of this genotype in this region. An interesting point to highlight is how this DTU diversity was able to maintain itself among these animals despite the low parasitemias. One explanation is that the triatomines presented the same haplotypes observed in the mammals, so they can be responsible for the transmission by being predated by the animals.

*Trypanosoma rangeli* lineage A and B infections were observed in *D. albiventris* and *R. rattus*. This is the first report of *T. rangeli* lineage B in the Caatinga, demonstrating that this lineage, together with lineage A (Dario et al., 2021b) presents a broad distribution in Brazil. *Didelphis albiventris* infection by *T. rangeli* lineage A was previously described (Dario et al., 2021b). We are adding one more rodent species infected by *T. rangeli* in Brazil, *R. rattus*. This new finding demonstrates that rodents with different ecological habits are capable of being infected with this species. *Trypanosoma dionisii* gains another mammalian host (*R. rattus*), proving to be a generalist species. *Trypanosoma lainsoni*, a species described in rodents (Naiff and Barrett, 2013), was observed in *D. albiventris* for the first time in the Caatinga biome; the first report on this species was in the Cerrado (Nantes et al., 2021), expanding its distribution spectrum. Here, we also report for the first time *R. rattus* infected by *C. mellificae*, a so-called monoxenous trypanosomatid. This species is increasingly dispersed among mammals in nature (Rangel et al., 2019; Alves et al., 2021; Dario et al., 2021a). This finding supports the likelihood that *C. mellificae* has a very eclectic vector or different vectors that make this species so widespread in Brazilian biomes, even in environments with a certain degree of degradation, as the infection was reported in a synanthropic animal.

Dogs presented high serological rates for *T. cruzi* infection in 2005 (Lima et al., 2012) and 2007 in the present study, but no parasitological exam yielded positive results. It is interesting that in Caatinguinha and Córrego das Melancias, locations where *T. cruzi* infection was detected in *R. rattus* and *D. albiventris*, the infection by *T. cruzi* was also reflected in domestic dogs, as most were infected. This observation confirmed the role of dogs’ sentinel host in the *T. cruzi* transmission cycle (Xavier et al., 2012), indicating the presence of the wild transmission cycle in the surroundings of the domestic environment.

In addition, the finding of so many ZOTUs/ASVs demonstrated that the *Trypanosoma* genus and within it, the *T. cruzi* clade, still offer unanswered questions. We highlight the following important questions here: What will be the impact of these mixed infections on the host in terms of their biological condition and infective potential? Are these ZOTUs/ASVs able to maintain themselves in the memory host or just in the vectors or the opposite? The same questions apply to *C. mellificae*, a monoxenous trypanosomatid that is increasingly found in subpatent infections in a broad spectrum of mammals. In conclusion, we can affirm that the Caatinga biome demonstrates remarkable trypanosomatid species/genotypes and haplotype richness. Two species are involved in *T. cruzi* enzootic transmission, *D. albiventris* and *R. rattus*, demonstrating that each animal has a different role in the transmission cycle (varies in different places and times) and therefore the importance of its investigation. The role of the dog in signaling the presence of *T. cruzi* was reinforced. Once again, there is much more to be unraveled than knowledge about trypanosomatids in nature given that new interactions and dispersions are observed. Thus, we are discovering a new world based on these findings.

## Data Availability Statement

The datasets presented in this study can be found in online repositories. The name of the repository and accession numbers can be found below: SRA, NCBI; PRJNA382386: SSR17675332 to SSR17675366.

## Ethics Statement

The animal study was reviewed and approved by Ethical Committee for Animal Use of the Oswaldo Cruz Foundation (P0179-03).

## Author Contributions

AJ, MD, SX, and CF contributed to the conception and design of the study. CL and SX organized the database. MD, CF, FS, CL, FO, and SX performed the research and analyses. MD and AJ wrote the first draft of the manuscript. All authors contributed to manuscript revision and read and approved the submitted version.

## Funding

This study was funded by the Fundação Oswaldo Cruz, Instituto Nacional de Câncer, Conselho Nacional de Desenvolvimento Científico e Tecnológico (CNPq), Coordenação de Aperfeiçoamento de Pessoal de Nível Superior (CAPES) and Fundaçao de Amparo a Pesquisa do Estado do Rio de Janeiro (Faperj). MD receives a postdoctoral fellow from Faperj (E-26/202.414/2019). FO receives a master’s grant from CAPES. FMS receives a postdoctoral fellow from CAPES (88887.162877/2018-00). AR is financially supported by CNPq/Universal (425293/2018-1) and Jovem Cientistas do Nosso Estado/Faperj (E-26/202.794/2019). SX received financial support from CNPq/Universal (422489/2018-2). AJ is financially supported by CNPq (Bolsista de Produtividade, nível 1A).

## Conflict of Interest

The authors declare that the research was conducted in the absence of any commercial or financial relationships that could be construed as a potential conflict of interest.

## Publisher’s Note

All claims expressed in this article are solely those of the authors and do not necessarily represent those of their affiliated organizations, or those of the publisher, the editors and the reviewers. Any product that may be evaluated in this article, or claim that may be made by its manufacturer, is not guaranteed or endorsed by the publisher.

## Supplementary Material

Supplementary Table 1 Kinetoplastid 18S rDNA sequence database.
